# Behaviour of Aquaporin Forward Osmosis Flat Sheet Membranes during the Concentration of Calcium-Containing Liquids

**DOI:** 10.3390/membranes10050108

**Published:** 2020-05-22

**Authors:** Alibek Omir, Aliya Satayeva, Aigerim Chinakulova, Arailym Kamal, Jong Kim, Vassilis J. Inglezakis, Elizabeth Arkhangelsky

**Affiliations:** 1 Department of Civil & Environmental Engineering, School of Engineering & Digital Sciences, Nazarbayev University, Nur-Sultan 010000, Kazakhstan; aomir@nu.edu.kz (A.O.); arailym.kamal@nu.edu.kz (A.K.); jong.kim@nu.edu.kz (J.K.); 2 Environmental Science & Technology Group (ESTg), Nazarbayev University, Nur-Sultan 010000, Kazakhstan; aliya.satayeva@nu.edu.kz (A.S.); aigerim.chinakulova@gmail.com (A.C.); vasileios.inglezakis@nu.edu.kz (V.J.I.); 3 National Laboratory Astana, Nazarbayev University, Nur-Sultan 010000, Kazakhstan; 4 Department of Chemical & Materials Engineering, School of Engineering & Digital Sciences, Nazarbayev University, Nur-Sultan 010000, Kazakhstan; 5 The Environment & Resource Efficiency Cluster (EREC), Nazarbayev University, Nur-Sultan 010000, Kazakhstan

**Keywords:** aquaporin, forward osmosis (FO), membrane, scaling, calcium

## Abstract

This study aims to examine the scaling and performance of flat sheet aquaporin FO membranes in the presence of calcium salts. Experiments showed that the application of calcium sulphate (CaSO_4_) resulted in an 8–78% decline in the water flux. An increase in the cross-flow velocity from 3 to 12 cm/s reduced the decline in the flux by 16%. The deposition of salt crystals on the membrane surface led to the alteration in the membrane’s intrinsic properties. Microscopy, attenuated total reflection-Fourier transform infrared (ATR-FTIR) spectroscopy, and X-Ray fluorescence (XRF) analyses confirmed measurements of the zeta potential and contact angle. The use of a three-salt mixture yielded severe scaling as compared with the application of calcium sulphate dehydrate (CaSO_4_ × 2H_2_O), i.e., a result of two different crystallisation mechanisms. We found that the amount of sodium chloride (NaCl), saturation index, cross-flow velocity, and flow regime all play an important role in the scaling of aquaporin FO flat sheet membranes.

## 1. Introduction

In the last decade, forward osmosis (FO) has attracted significant attention globally. FO is driven by osmotic pressure and, as a result, requires less energy than conventional pressure-driven membrane separation processes. In spite of this advantage, the industrial applications of the FO process are still limited. For example, FO is a component of the desalination process used at the Al Khaluf treatment plant in Oman, which has a capacity of 200 m^3^/day [1]. The plant uses FO to dilute the draw solution before it is desalinated by reverse osmosis. The diluted reverse osmosis feed decreases the desalination energy requirements by more than 20% [2]. Another example of an FO process industrial application is at the Statkraft prototype plant in Norway, which used river and seawater to generate power. Plant operation began in 2009 and terminated in 2014 due to the limited salinity gradient between river and seawater, as well as the membrane permeability [3]. Such a failure has led to the development of a new type of filtration media, i.e., membranes with embedded aquaporin. Aquaporin is a specialised class of proteins, which facilitates water transport across a membrane in living organisms. The concept of FO with embedded aquaporin was first proposed in 2012 [4]. A limited number of early studies did not report detailed experimental results. For example, Nielsen [5] briefly presented a general strategy for biomimetic membranes. Several research groups have attempted to apply this approach; however, a conceptual demonstration has remained a challenge [6,7,8,9].

Although FO membranes are less susceptible to fouling, they still suffer from an accumulation of foulants. As aquaporin FO is a new technology, there are currently a limited number of studies focused on fouling. For example, Hey et al. [10] revealed that the pre-treatment of raw municipal wastewater affects the degree of fouling in biomimetic FO membranes. Li et al. [11] showed that the aquaporin FO membrane maintains a stable flux of approximately 5 L/m^2^-h (LMH) for 16 days, with the application of municipal secondary wastewater effluent. Song et al. [12] performed experiments on an FO-membrane distillation system, reporting that the application of dairy wastewater may impair the permeability of aquaporin FO membranes both gradually and sharply immediately after the application of the feed solution. Concentrations of fumaric acid, with an L-alanine draw solution, yielded a sharp decline in the flux within 4 h [13]. Xue et al. [14] bound TiO_2_ nanoparticles to aquaporin FO membranes and detected an antifouling affect for an organic feed solution. Luo et al. [15] applied an aquaporin FO membrane in an osmotic membrane bioreactor, observing a gradual decline in the flux of synthetic wastewater. Singh et al. [16] analysed concentrations of molasses distillery wastewater with a biomimetic membrane, finding that the critical water flux and critical draw solution are below 4 LMH and 3 M (MgCl_2_ × 6H_2_O), respectively. When Soler-Cabezas et al. [17] examined anaerobically digested sludge concentrations via aquaporin FO, they detected anomalous behaviours in the membrane after approximately 50 h of filtration before observing a sharp decrease and subsequent plateau in the water flux. Soler-Cabezas et al. [17] attributed such behaviour to the formation of a cake layer on the membrane surface. The aquaporin water channels were apparently blocked by precipitated salts or organic matter, which were later back-transported to the bulk solution with the recovery of the water flux. Kalafatakis et al. [18] applied the membranes to the fermentation of glycerol, detecting an approximately 90% decline in the water flux at a 5 cm/s cross-flow velocity over 15 h. Munshi et al. [19] investigated the dewatering of algae, observing that a NH_4_Cl draw solution is the best candidate for an improved water flux and low reverse salt flux, such that the increase in cross-flow velocity (2–11 cm/s) may enhance the permeate flux by 5–10%. From the works listed in this paragraph, it can be seen that all investigations were concentrating on organic fouling with a focus on the water flux and retention capability of the aquaporin FO membranes. Fouling mitigation or the efficiency of membrane cleaning methods were barely studied. Hence, research dedicated to other types of fouling with an emphasis on an understanding of its mechanisms, the influence of different parameters on the process productivity, the efficiency of the process and the membrane cleaning techniques are of high importance.

Previous studies focused on aquaporin activity have shown that there may be inhibited water transport through aquaporins. Preston at al. [20] and Barone at al. [21] found that mercury causes either a blockage or conformational change in the protein, which leads to inhibited water transport. Niemietz and Tyerman [22] tested aquaporins such as NOD 26, plasma membrane integral protein, and human aquaporin 1, observing that silver and gold, as AgNO_3_, silver sulfadiazine, or HAuCl_4_, can nearly fully inhibit water permeability. Other elements, such as cobalt, copper, cadmium, nickel, zinc, lanthanum, barium, lead, and platinum, may also significantly suppress the permeability of water through the protein channels. Martınez-Ballesta et al. [23] reported aquaporin closure triggered by cytosolic calcium and salt stress as an inhibitory mechanism at aquaporin level, and up-regulation of aquaporins by calcium at the whole plant level.

Calcium is one of the main constituents in sea and wastewater. During treatment, calcium concentrations may reach elevated levels. An exceedingly high solubility level leads to crystal formation and, as a result, membrane scaling, where the crystallisation mechanism can possibly have a drastic effect on membrane performance. For example, Shih et al. [24] reported that both bulk and surface crystallisation controls the crystallisation of calcium sulphate (CaSO_4_) (i.e., a mix of calcium chloride (CaCl_2_), magnesium sulphate (MgSO_4_), and sodium sulphate (Na_2_SO_4_)). Lin and Cohen [25], Xie and Gray [26], and Shaffer et al. [27] reported that a surface crystallisation mechanism governs gypsum scaling on a polyamide membrane. However, bulk crystallisation mechanisms have been reported for cellulose acetate membranes [26,28]. Furthermore, previous studies have shown that bulk crystallisation has less of a negative effect on membrane performance than surface crystallisation.

Based on the authors’ knowledge, this is the first systematic and controlled study on aquaporin FO membrane scaling. Aspects such as the influence of draw solution concentration on the process productivity, contribution of each salt to the degree of scaling, effect of saturation index, nature of calcium-containing liquid, cross-flow velocity, and direction of pumping were studied for the first time. We examined the effect that the crystallisation mechanism has on the water flux by applying (i) a mixture of sodium chloride (NaCl), CaCl_2_, and Na_2_SO_4_ and (ii) calcium sulphate dihydrate (CaSO_4_ × 2H_2_O). Experiments were conducted in an active layer facing feed solution (AL-FS) configuration. The membranes were characterised before and after the scaling experiments.

## 2. Materials and Methods

### 2.1. Membrane and Chemicals

A commercially available aquaporin FO flat sheet membrane was used in this study. The membrane was a thin film composite with embedded protein in the active layer (Aquaporin A/S, Kongens Lyngby, Denmark). The membrane had a thickness of 110 microns, consisting of a polyamide active layer and polyethersulphone support. NaCl, CaCl_2_, Na_2_SO_4_, and CaSO_4_ × 2H_2_O were purchased from Sigma–Aldrich, St. Louis, MO, USA. Milli-Q water (Integral 15, Merck, Darmstadt, Germany) was used for the preparation of all solutions. 

### 2.2. FO Experiment

The FO setup used in this study was described earlier and included two variable-speed peristaltic pumps (Cole-Parmer, Vernon Hills, IL, USA), membrane holder (Sterlitech, Kent, WA, USA), electronic balance (OHAUS, Parsippany, NJ, USA), and stirrer plate [29]. The membrane holder was aligned horizontally, the draw solution was placed on the balance, and the feed on the stirrer plate. The pumps were used to circulate the feed and draw side streams through the membrane holder with a channel depth of 2.3 mm and effective filtration area of 4 × 8.5 cm^2^. The feed and draw side streams were pumped at identical cross-flow velocities through both sides of the membrane. A spacer was used to support the membrane. The experiments were conducted at ambient temperature.

FO experiments were performed for 6 h. NaCl (1–5 M) was used as the draw solution. Tests with a 10 mM NaCl feed solution were performed to evaluate FO flux behaviour without the presence of scalants. To prepare the feed solutions with different CaSO_4_ saturation indices (SIs), varying salt concentrations were used. Table 1 lists the detailed compositions of the feed solutions used for the scaling experiments. Unless otherwise specified, the following reference conditions were applied to all scaling experiments: an AL–FS orientation; 12.5 cm/s cross-flow velocity; initial water flux of 13 LMH; and the feed and draw solutions were circulated counter-currently. A digital balance was used to record the water flux at predetermined time intervals. The water flux values were normalized to the initial water flux for the scaling experiments.

The experiments were replicated to ensure the reproducibility of results. The water flux profiles were plotted by taking the average values obtained from replicate FO experiments.

### 2.3. Membrane Characterisation Methods

Both pristine and scaled (SI 2.45) membranes were characterised. A SurPASS electrokinetic analyser (Anton Paar GmbH, Graz, Austria) was used to determine the zeta potential of the membrane. Here, 1, 10, and 100 mM potassium chloride (KCl) solutions were pumped through an adjustable gap sample holder. The streaming potential was detected via Ag/AgCl electrodes located at both ends of the sample. Measurements were performed at pH range of 2–11. The pH of the electrolyte was adjusted using either a 0.1 M KOH or HCl solution. Measurements of the contact angle were performed following the standard protocol: a drop of water was placed onto the membrane surface using a syringe and the air–water–surface contact angle was measured within 10 s [30]. The Leica DM 500 optical microscope (Leica Microsystems, Wetzlar, Germany) and an FESEM Auriga 50 scanning electron microscope (SEM) were used to characterise the membrane’s surface morphology. Before SEM imaging, samples were coated with a layer of gold using a Q150T automatic sputter coater. Fourier transform infrared (FTIR) analysis was performed with a Cary 660 FTIR spectrometer (Agilent, Santa Clara, CA, USA), combined with an attenuated total reflectance (ATR) device. The ATR-FTIR analysis was used to study the chemical nature of both pristine and scaled membranes. An Axios mAX X-ray fluorescence (XRF) (Malvern Pananalytical, Malvern, U.K.) was used to determine the elemental composition of the membranes. All measurements were performed a minimum of three times.

## 3. Results and Discussion

### 3.1. Performance of the Membrane without Scalants

**Figure 1:**
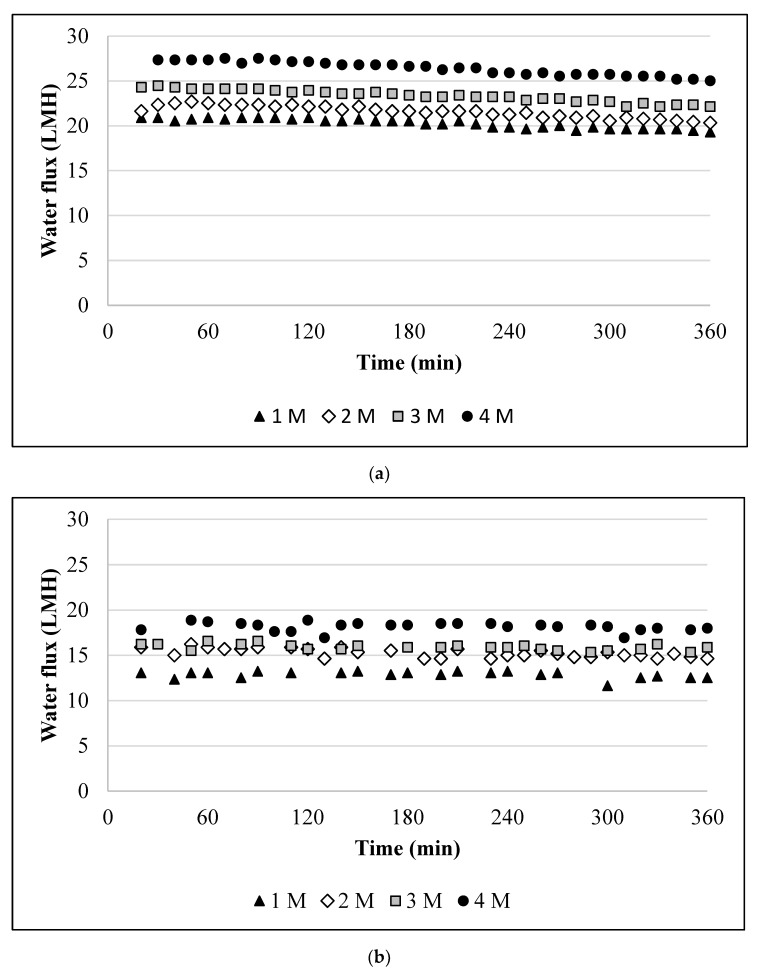
The water flux of the membrane in the absence of scalants: (**a**) active layer-facing draw solution (AL-DS) orientation; and (**b**) AL-feed solution (FS) orientation. Experimental conditions: concentrations of NaCl, CaCl_2_, and Na_2_SO_4_ in the feed are 10, 0, and 0 mM, respectively; 12.5 cm/s cross-flow velocity; and the feed and draw solutions were circulated counter-currently.

**Figure 2:**
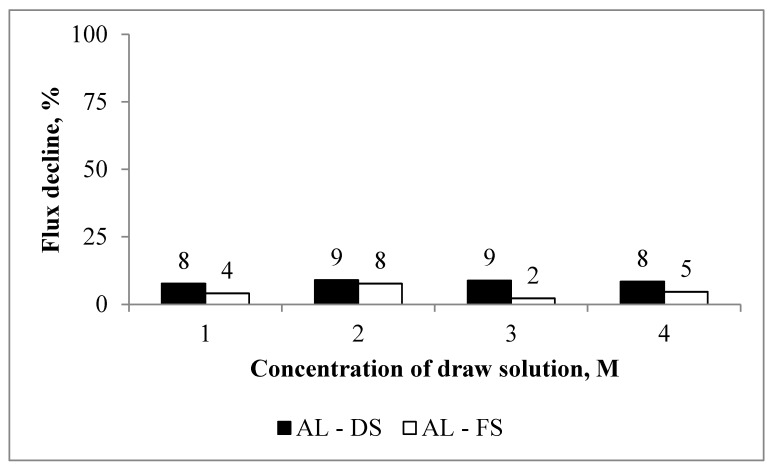
The decline in the flux observed for the baseline experiments. Experimental conditions: concentrations of NaCl, CaCl_2_, and Na_2_SO_4_ in the feed are 10, 0, and 0 mM, respectively; 12.5 cm/s cross-flow velocity; and the feed and draw solutions were circulated counter-currently.

To differentiate concentration-polarisation, dilution of the draw solution, and the scaling effect, the performance of the membrane was first examined without scalants. Figure 1 depicts the water flux as a function of the draw solution concentration in different orientations. The figure shows that, for identical draw solution concentrations, the water fluxes in the AL-FS were lower than those in the active layer facing draw solution (AL-DS) configuration. For example, for a 4 M draw solution, the water flux in the AL-FS was 1.5-fold lower than in the AL-DS (17.82 vs. 27.33 LMH). We did not observe a significantly higher decline in the water flux (Figure 2) for the AL-DS orientation. 

### 3.2. Membrane Behaviour in the Presence of Scaling Solutions

**Figure 3:**
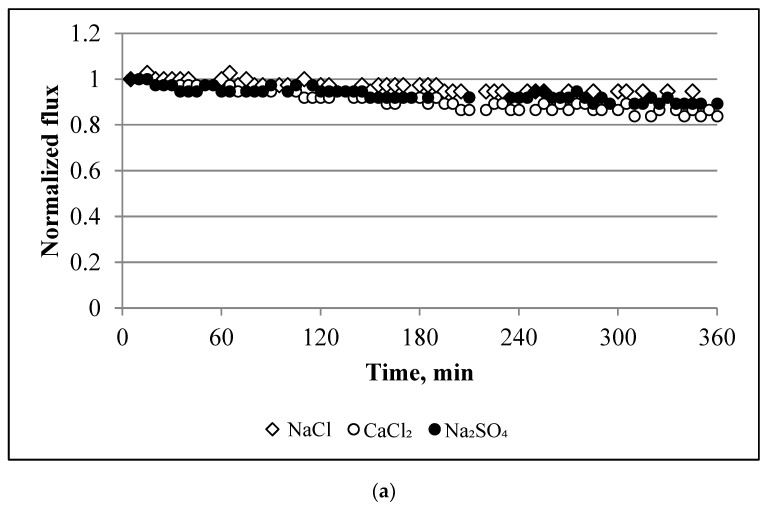

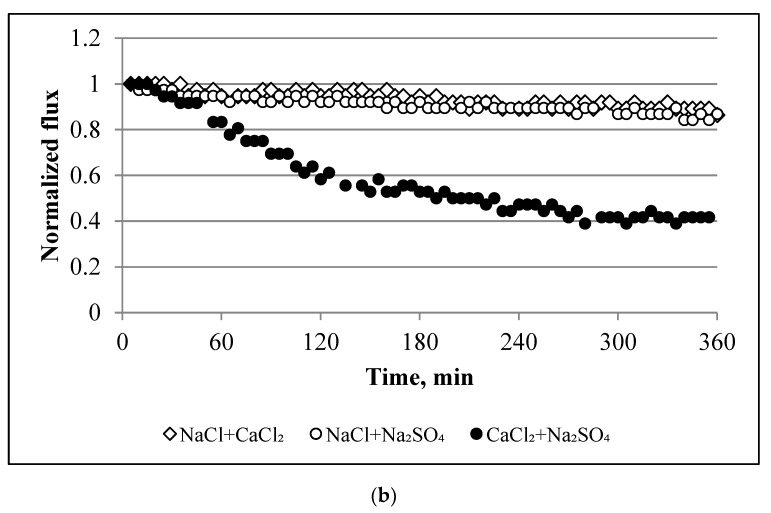
Normalized water flux profiles during the filtration of (**a**) single salt and (**b**) mix of two salts. Experimental conditions: concentrations of NaCl, CaCl_2_, and Na_2_SO_4_ are 1.754, 6.132, and 4.487 g/L, respectively; 12.5 cm/s cross-flow velocity; the feed and draw solutions were circulated counter-currently; and an AL-FS orientation.

**Figure 4:**
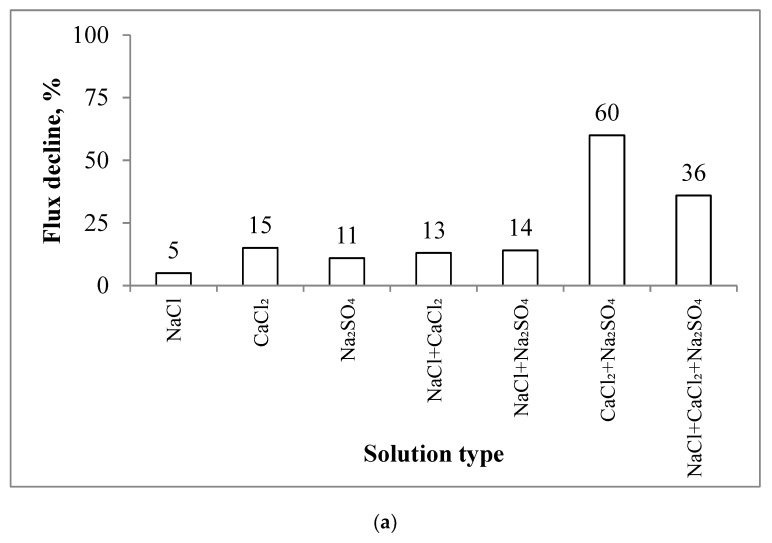

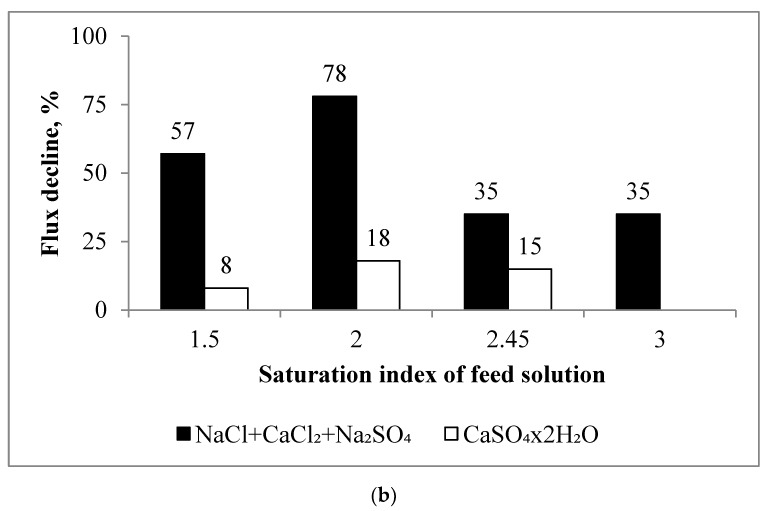
The decline in the flux observed for the scaling experiments: (**a**) single, binary, and ternary feed solutions; and (**b**) at different saturation indices for NaCl + CaCl_2_ + Na_2_SO_4_ and CaSO_4_ × 2H_2_O. Experimental conditions: 12.5 cm/s cross-flow velocity; the feed and draw solutions were circulated counter-currently; and an AL-FS orientation.

The investigation of scaling in the aquaporin FO flat sheet membranes began based on a study of the water fluxes for the single salts, i.e., NaCl, CaCl_2_, and Na_2_SO_4_. Figure 3a and Figure 4a show the results obtained from these experiments. The application of single salts to the membrane resulted in a minor reduction in the flux. For example, the decline in the flux was 5%, 15%, and 11% for NaCl, CaCl_2_, and Na_2_SO_4_, respectively. When the salts were mixed to form binary solutions, the flux decreased by 13–14% for NaCl+CaCl_2_ and NaCl + Na_2_SO_4_ and 60% for CaCl_2_ + Na_2_SO_4_ (Figure 3b and Figure 4a). 

**Figure 5:**
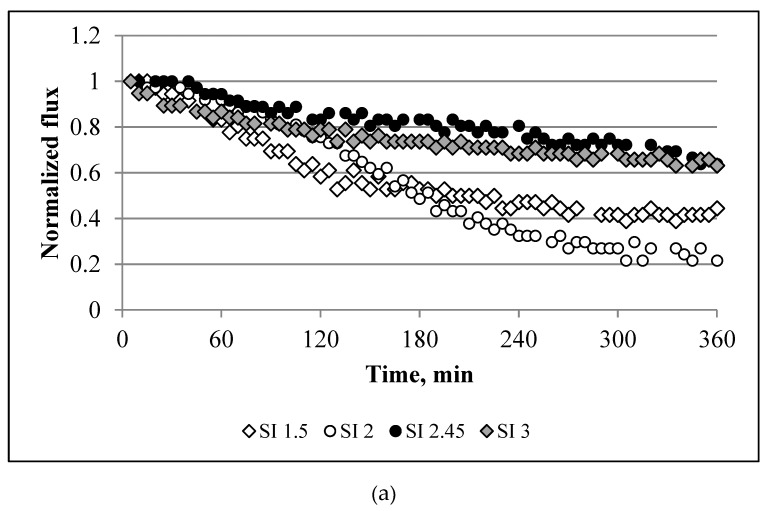

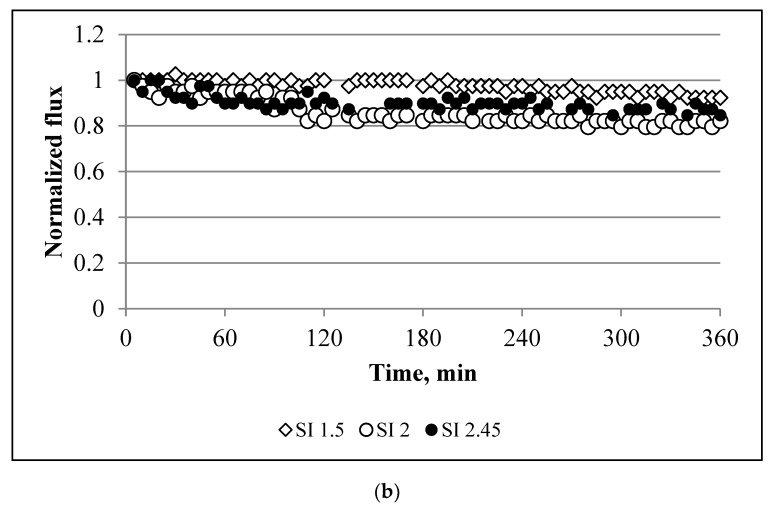
Normalized flux for: (**a**) NaCl + CaCl_2_ + Na_2_SO_4_; and (**b**) CaSO_4_ × 2H_2_O at different saturation indices (Table 1 lists the concentrations of the salts). Experimental conditions: 12.5 cm/s cross-flow velocity; the feed and draw solutions were circulated counter-currently; and an AL-FS orientation.

Figure 4 and Figure 5a show the water flux profiles for a mixture of three salts at different saturation indices. The largest decline in the water flux was observed for SIs of 1.5 and 2. The decrease in the water flux for these cases were 57% and 78%, respectively. An increase in SI of up to 2.45 and 3 suppressed the decline in the flux by up to 35%. The experiments performed with NaCl + CaCl_2_ + Na_2_SO_4_ were also compared with CaSO_4_ × 2H_2_O (Figure 4b and Figure 5b). The effect of CaSO_4_ × 2H_2_O on membrane performance was less severe than that based on the mix of the three salts. All CaSO_4_ × 2H_2_O SIs showed a range from 8–18% for a decline in the water flux.

**Figure 6:**
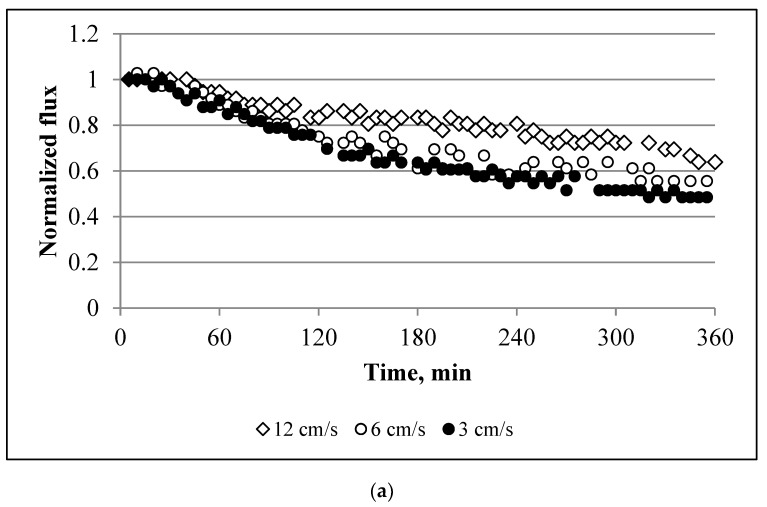

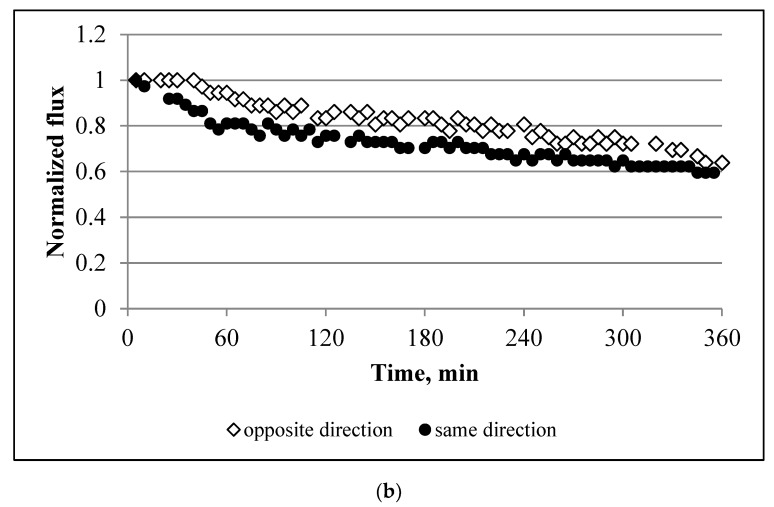
Effect of the: (**a**) cross-flow velocity; and (**b**) feed/draw solution pumping direction on the normalized flux. Experimental conditions: concentrations of NaCl, CaCl_2_, and Na_2_SO_4_ are 1.754, 6.132, and 4.487 g/L, respectively; SI = 2.45; and an AL-FS orientation.

**Figure 7:**
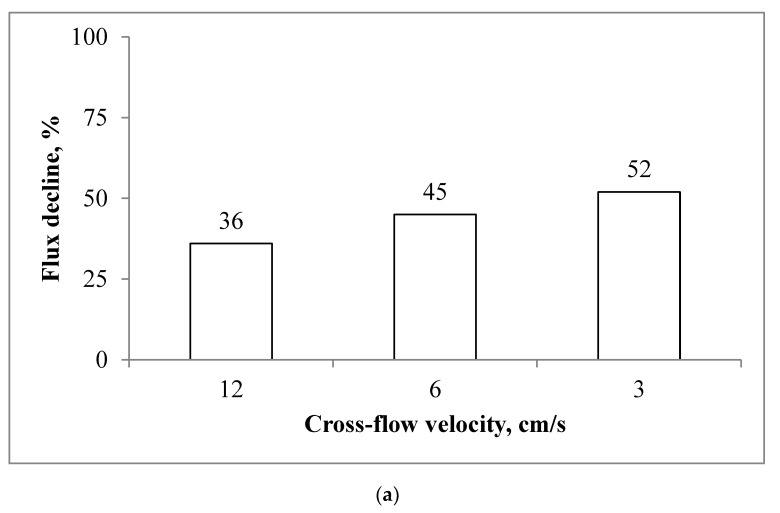

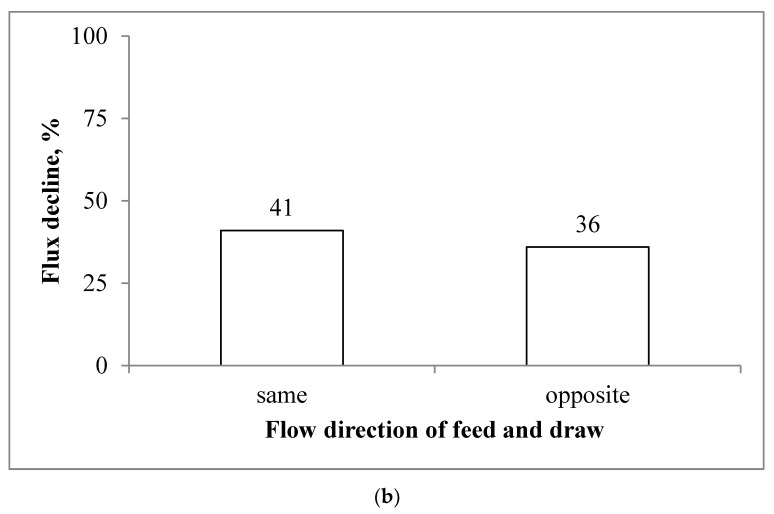
The decline in the flux observed for the scaling experiments: (**a**) effect of the cross-flow velocity; and (**b**) influence of the flow direction. Experimental conditions: concentrations of NaCl, CaCl_2_, and Na_2_SO_4_ are 1.754, 6.132, and 4.487 g/L, respectively; SI = 2.45; and an AL-FS orientation.

**Figure 8:**
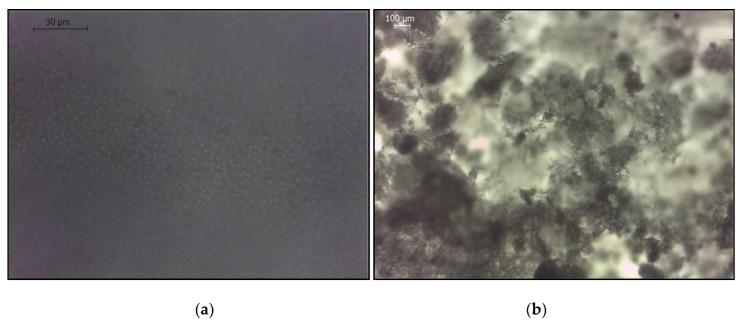

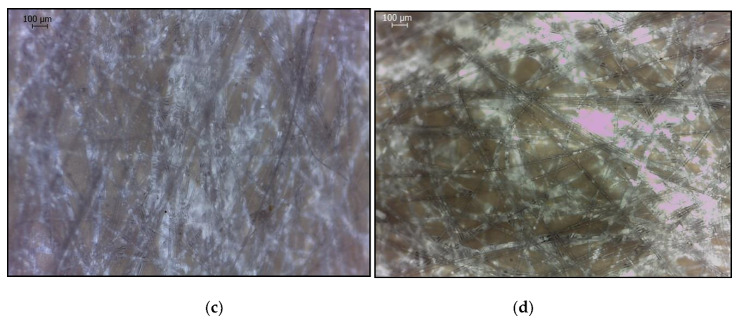
Optical microscope images of the membrane: (**a**) active layer before scaling; (**b**) active layer after scaling; (**c**) support layer before scaling; and (**d**) support layer after scaling. Scaling conditions: concentrations of NaCl, CaCl_2_, and Na_2_SO_4_ are 1.754, 6.132, and 4.487 g/L, respectively; SI = 2.45; 12.5 cm/s cross-flow velocity; the feed and draw solutions were circulated counter-currently; and an AL-FS orientation.

Figure 6a and Figure 7a depict the influence that the cross-flow velocity has on membrane performance. A reduction in the cross-flow velocity from 12.5 to 3 cm/s intensifies the decline in the water flux, i.e., at 12.5, 6, and 3 cm/s the water flux at the end of experiment was equal to 64%, 55%, and 48%, respectively. Figure 7b and Figure 8b show the effect of the feed and draw solution pumping direction. The membrane yielded a slightly higher average water flux in counter-current flow mode.

### 3.3. Characterisation of the Pristine and Scaled Membranes

**Figure 9:**
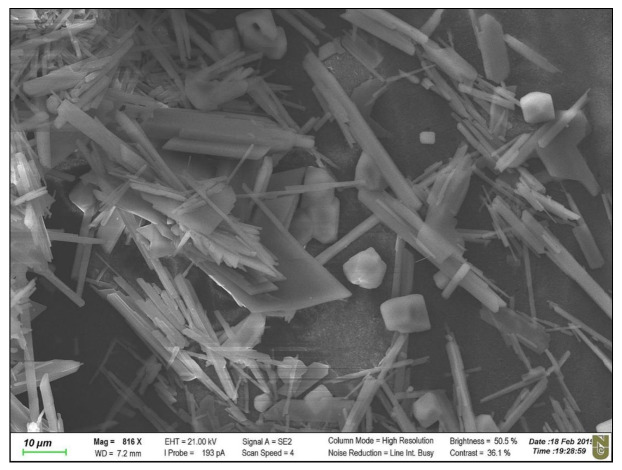
SEM image of the aquaporin FO membrane’s active layer after the scaling experiment. Scaling conditions: concentrations of NaCl, CaCl_2_, and Na_2_SO_4_ are 1.754, 6.132, and 4.487 g/L, respectively; SI = 2.45; 12.5 cm/s cross-flow velocity; the feed and draw solutions were circulated counter-currently; and an AL-FS orientation.

After the application of the SI 2.45 solution (mixture of three salts), the membrane was unevenly covered by crystals (based on a visual analysis). Figure 8 shows the scaled areas of the active layer and support layer after the FO experiment, comparing these areas with the intact membrane (based on an optical microscope analysis). We can observe that, after the FO experiment, the support layer had not changed, whereas certain parts of the active layer were fully covered by CaSO_4_ crystals. Figure 9 shows that crystals, formed during the application of NaCl + CaCl_2_ + Na_2_SO_4_ (SI = 2.45), accumulated on the active layer of the membrane, and the crystal sizes were significantly larger than the membrane pore size [31].

The hydrophobicity of the membrane was evaluated using the contact angle method. The contact angles of the active and support layers for the pristine membrane were 53° and 61°, respectively. These values suggest that both layers are hydrophilic, while the support layer is less hydrophilic than the active layer. When the membrane was exposed to the feed (three salts with SI = 2.45), there was a reduction in these values. The contact angle of the active layer was 41°, while the contact angle of the support layer was 53°. These results indicate that the accumulation of substances on the membrane’s surface leads to increased membrane hydrophilicity.

**Figure 10:**
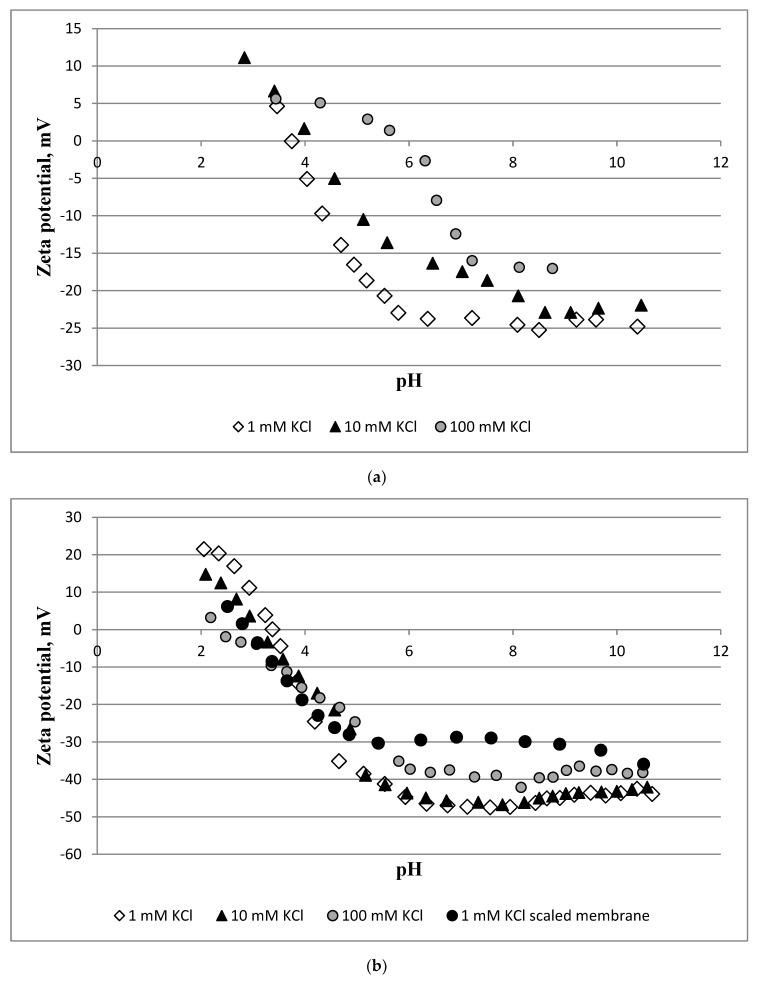
The zeta potential of the membrane: (**a**) support layer; and (**b**) active layer at 1, 10, 100, and 1 mM KCl scaled membrane. Scaling conditions: concentrations of NaCl, CaCl_2_, and Na_2_SO_4_ are 1.754, 6.132, and 4.487 g/L, respectively; SI = 2.45; 12.5 cm/s cross-flow velocity; the feed and draw solutions were circulated counter-currently; and an AL-FS orientation.

Figure 10 shows the zeta potential of the membrane for a pH range from 2–11. 

Comparing the intact active and support layers, we can observe that the former is characterised by more negative values. For example, from a pH of 4 to 11, the zeta potential of the active and support layers was equal to −24.6 to −42.1 mV and −5.1 to −24.8 mV (in a 1 mM KCl solution), respectively. The polyamide isoelectric point was detected at approximately a pH of 3 while polyethersulphone was detected between a pH of 4 and 6. The zeta potential of both the support and active layers is more positive at higher concentrations of KCl and lower pH values. The exposure of the membrane to the scaling solution (NaCl + CaCl_2_ + Na_2_SO_4_, SI = 2.45) altered the membrane’s charge, i.e., the active layer became more positive. In a 1 mM KCl solution, the scaled membrane showed a 2.5 to −35.9 mV range in the zeta potential.

Table 2 lists the elemental compositions of the membrane from XRF analyses.

Since the concentration of carbon, nitrogen, and oxygen could not be taken into account, Table 2 lists the normalized sum of the detected elements, i.e., 100% (the actual sum of the detected elements is 29%) [32]. Table 2 indicates that sulphur is the main elemental constituent in the virgin membrane (87%). The chlorine, titanium, and calcium contents ranged between 2% and 5%. Other elements, such as potassium, iron, silicon, magnesium, nickel, copper, and zinc, were also detected in the membrane, but their content was insignificant, i.e., less than 1%. XRF analysis of the scaled membrane indicates that the percentage of sulphur reduced to 30% while calcium increased to 64%. The concentration of chlorine increased by 2.3% and the amount of titanium, iron, magnesium, and nickel was less than 1%. 

**Figure 11:**
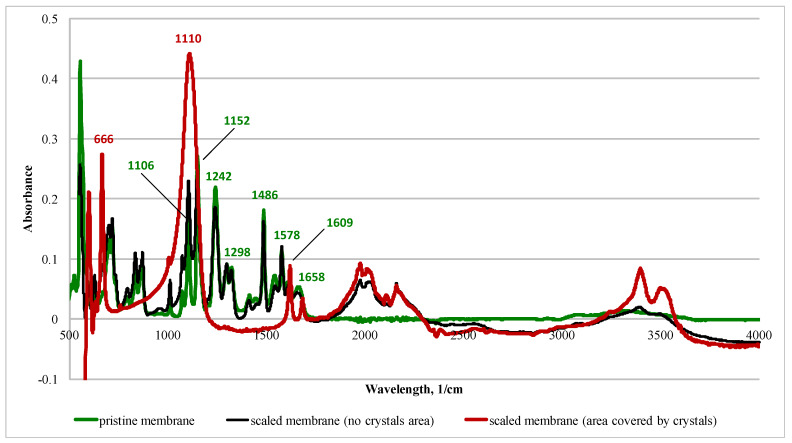
ATR-FTIR spectra of the membranes. Scaling conditions: concentrations of NaCl, CaCl_2_, and Na_2_SO_4_ are 1.754, 6.132, and 4.487 g/L, respectively; SI = 2.45; 12.5 cm/s cross-flow velocity; the feed and draw solutions were circulated counter-currently; and an AL-FS orientation.

Figure 11 shows the ATR-FTIR spectra of the membranes.

The pristine membrane is characterised by a polyamide characteristic peak at 1578 cm^−1^ (C–N stretching, amide II), 1609 cm^−1^ (–N–H), and 1658 cm^−1^ (C=O stretching, amide I) [10]. For the polyethersulphone support, peaks were observed at 1486 cm^−1^, 1298 cm^−1^ (SO_2_, asymmetric stretch), 1242 cm^−1^ (aryl–O–aryl, C–O stretch), 1152 cm^−1^ (SO_2_, symmetric stretch), and 1106 cm^−1^ (skeletal aliphatic C–C/aromatic hydrogen bending/rocking) [18]. Areas of the membrane where the crystals were not visually observed (after scaling) had similar spectra. In contrast, the membrane area that was covered by crystals exhibited different a spectrum, i.e., strong peaks were observed at 1110 cm^−1^ and 666 cm^−1^ (see the following section).

### 3.4. Discussion

Fick’s law states that the rate of transfer of molecules or atoms via diffusion through a unit area is proportional to the concentration gradient [33]. The baseline experiments show (Figure 1) that there was an initial increase in the water flux, which was not proportional to the concentration of the draw solution. For example, the initial water flux for the AL-DS was 27, 24, 22, and 21 LMH for NaCl concentrations of 4, 3, 2, and 1 M, respectively. In contrast, the AL-FS orientation had water fluxes of 18, 16, 16, and 13 LMH for identical concentrations to the draw solution. This behaviour can be attributed to the internal concentration polarisation (ICP) effect. Higher values of the water flux in the AL-DS orientation were due to a concentrative ICP effect, which is lower than a dilutive ICP effect (as was the case for the AL-FS orientation). Based on the results, we also observe that the flux was more stable in the AL-FS orientation. This is due to the low water flux and severe dilutive ICP effect in the AL-FS orientation. The internal ICP in the AL-DS orientation is a product of accumulation of ions inside of the membrane’s support layer at the time of filtration. On the other hand, the internal ICP in the AL-FS configuration is arising from dilution of the draw solution by the permeate inside of the support layer. Both the internal ICP in the AL-DS and the AL-FS lead to a net driving force decrease. The water flux profiles obtained for the baseline experiments are similar to those reported for FO membranes without aquaporin [34].

By comparing the baselines with the scaling experiments, we observe that the reduction in the water flux was mainly caused by scaling, i.e., not due to the dilution of the draw solution. The dilution of the draw solution resulted in only a 2–8% decline in the water flux. Using the results obtained for the single salts (Figure 4a) and applying the additive flux concept, we can calculate the decline in the flux decline for a mix of two or three salts. For example, the calculated decline in the water flux decline for NaCl + CaCl_2_ is 20%, 26% for CaCl_2_ + Na_2_SO_4_, 16% for NaCl + Na_2_SO_4_, and 31% for three combined salts. The decline in the water flux caused by the NaCl solution was 5%. By comparing this result with the baseline experiments, the decline in the water flux from NaCl was only due to the dilution of the draw solution. Comparing the calculated additive flux values with the experimental data, we can observe that only one solution, i.e., CaCl_2_ + Na_2_SO_4_, has a significant difference between the calculated and experimental values. The calculated value was 2.3-fold lower than the value obtained from the experiments (26% vs. 60%). For the other solutions, this difference was minor, i.e., 13.5 vs. 20 LMH for NaCl + CaCl_2_, 13.5 vs. 16 LMH for NaCl + Na_2_SO_4_, and 35 vs. 31 LMH for the three salts. The NaCl + CaCl_2_ + Na_2_SO_4_ feed solution had a weaker decline in the flux than CaCl_2_ + Na_2_SO_4_ (35% vs. 60%). This is because, as NaCl was introduced into the feed, an increase in the ionic strength led to a reduction in the ionic activity of calcium and sulphate, which resulted in incremental solubility and a decrease in the saturation degree, otherwise known as the “salt in” effect [35]. This also explains the severe decline in the flux for SI = 1.5 and 2 as compared with the solutions at an SI of 2.45 and 3 (i.e., a higher concentration of NaCl increased the solubility). However, experiments performed with CaSO_4_ × 2H_2_O were characterised by a negligible decline in the flux (for all saturation indices) compared with the NaCl + CaCl_2_ + Na_2_SO_4_ solution (Figure 4). This is because bulk crystallisation controls the scaling of CaSO_4_ × 2H_2_O while surface crystallisation dominates the scaling of CaSO_4_. Surface crystallisation results in more a severe decline in the flux than bulk crystallisation [28].

The experiments performed at different cross-flow velocities show that membrane performance can be improved by an increase in the speed of the draw and feed solution pumping (Figure 6a). This indicates that a higher shear rate tends to mitigate scaling by hindering the deposition of the scalant on the membrane. These findings agree with previous studies [36]. However, a two-fold increase in the cross-flow velocity does not proportionally suppress scaling. For example, an increase in the cross-flow velocity from 3 to 6 cm/s mitigates water flux reductions by 13%, and by 20% for 6 and 12 cm/s. These results should be considered for further process optimisation and energy savings. The flat sheet aquaporin membrane used in this study also showed that the direction of pumping has a negligible effect on the membrane performance (Figure 6b). Improved membrane performance in a counter-current regime may be related to the net driving force, which is lower at the outlet point of the FO module in the co-current configuration [37,38].

A recent study [39] demonstrated that the water flux for an aquaporin FO membrane (Aquaporin A/S, Denmark) decreased from 20 to 15 LMH within 10 h of filtration. By comparing the results reported in Chun et al. [39] with data presented here, we can observe that similar SIs (i.e., 1.5 vs. 1.3) showed a 60 (current study) and 25% Chun et al. (Chun et al., 2018) decline in the water flux. Similar to this study, Chun et al. [39] performed FO experiments in the AL-FS orientation and counter-current mode, where NaCl was used as a draw solution for the scaling experiments. In contrast, the initial water flux and cross-flow velocity were 20 LMH and 9.5 cm/s (Chun et al., 2018), respectively. Such differences in experimental results can be explained by non-consistent characteristics of the membrane across batch production. This assumption may be supported by the current unavailability of aquaporin FO flat sheet membranes from Aquaporin A/S (Denmark).

**Figure 12:**
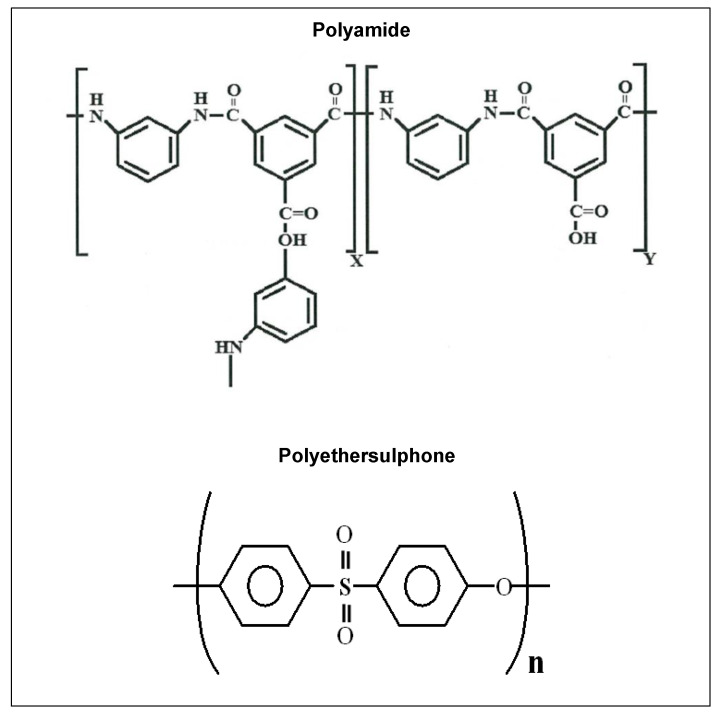
Chemical structure of the polymers.

The zeta potential, contact angle, microscopy, ATR-FTIR, and XRF analyses confirmed that the membrane was scaled during the FO experiments. The zeta potential measurements showed that the membrane charge increases with an increase in the KCl concentration. Elevated KCl concentrations led to a shrinking of the electric double layer, yielding a reduced zeta potential value. Changes in the membrane charge with the pH are due to the ionisation of carboxyl groups in the polyamide active layer (Figure 12), and a result of anion (Cl^−^) adsorption from the electrolyte to a polyethersulphone support [40].

The scaling layer on the membrane surface affected the charge of the active layer. For example, at a neutral pH, the membrane zeta potential increased from −47 to −29 mV. This is a result of the deposition of positively charged crystals [41]. Salt deposition on the membrane also elevated the hydrophilicity of both the active and support layers. This can be attributed to the presence of salt crystals that are hydrophilic in nature [42]. Here, 87% of the sulphur detected by XRF is from sulphur in the polyethersulphone support layer (Figure 12). When the scaling solution was applied to the membrane, calcium became a dominant element. Strong peaks observed at 1110 and 666 cm^−1^ are the characteristic peaks in the sulphate [43,44].

## 4. Conclusions

This study aimed to investigate the effect of calcium-containing salts on water transport through aquaporin channels and scaling in the aquaporin FO flat sheet membranes. The application of the scaling solutions resulted in the alteration in the membrane’s intrinsic properties. Similar to other FO membranes, the membranes with embedded aquaporin are susceptible to concentration polarisation. The membrane’s exposure to the feed solution containing only calcium ions (CaCl_2_ feed solution) resulted in a 15% decline in the water flux while a mix of the three salts led to a 35% reduction in the flux. This indicates that the decrease in the water flux occurred due to the membrane scaling, i.e., not the inhibition of the aquaporin channels. The highest degree of scaling was associated with the SI = 2 feed solution, which is a result of the “salt out” effect. Our results suggest that bulk crystallisation mechanisms are more favourable for the aquaporin membranes. The process performance can be enhanced by the optimisation of both the feed- and process-related parameters, i.e., the pre-treatment of the feed solution, as well as the adjustment of the cross-flow velocity and flow regime.

## Figures and Tables

**Table 1:** Compositions of the feed solutions used in this study.

	Feed Solution (g/L)			
SI	NaCl	CaCl_2_	Na_2_SO_4_	CaSO_4_ × 2H_2_O
*Multi-component feed*				
1.5	1.188	4.153	3.039	
2	1.493	5.22	3.82	
2.45	1.754	6.32	4.487	
3	2.138	7.475	5.469	
*Single-component feed*				
1.5				2.756
2				3.47
2.45				4.078

**Table 2:** XRF analyses of the intact and scaled aquaporin forward osmosis (FO) flat sheet membrane.

	Concentrations (wt. %)	
*Element*	*Intact*	*Scaled*
S	87.47	29.58
Cl	4.27	6.56
Ti	3.57	0.43
Ca	2.97	63.59
K	0.46	---
Fe	0.42	0.17
Si	0.15	---
Mg	0.13	0.09
Ni	0.10	0.05
Cu	0.07	---
Zn	0.03	---
